# Inadvertent Administration of 72 µg of Follitropin-Δ for Three Consecutive Days Does Not Appear to Be Dangerous for Poor Responders: A Case Series

**DOI:** 10.3390/jcm12165202

**Published:** 2023-08-10

**Authors:** Giorgio Maria Baldini, Antonella Mastrorocco, Romualdo Sciorio, Simone Palini, Miriam Dellino, Eliano Cascardi, Gerardo Cazzato, Antonio Malvasi, Domenico Baldini, Giuseppe Trojano

**Affiliations:** 1 IVF Center, Momo Fertilife, 76011 Bisceglie, Italy; gbaldini97@gmail.com; 2 Department of Bioscience Biotechnologies and Environment, University of Bari “Aldo Moro”, 70125 Bari, Italy; antonella.mastrorocco@uniba.it; 3 Edinburgh Assisted Conception Programme, Royal Infirmary of Edinburgh, Edinburgh EH16 4SA, UK; sciorioromualdo@hotmail.com; 4 Department of IVF, “San Giorgio” Hospital—AUSL Romagna, 47841 Cervia, Italy; simonepalini@yahoo.it; 5 Obstetrics and Gynecology Unit, Department of Biomedical Sciences and Human Oncology, University of Bari “Aldo Moro”, Piazza Giulio Cesare 11, 70124 Bari, Italy; miriamdellino@hotmail.it (M.D.); antoniomalvasi@gmail.com (A.M.); 6 Department of Medical Sciences, University of Turin, 10124 Turin, Italy; eliano.cascardi@ircc.it; 7 Pathology Unit, FPO-IRCCS Candiolo Cancer Institute, 10060 Candiolo, Italy; 8 Section of Molecular Pathology, Department of Emergency and Organ Transplantation (DETO), University of Bari “Aldo Moro”, 70124 Bari, Italy; gerardo.cazzato@uniba.it; 9 Department of Maternal and Child Health, Madonna delle Grazie Hospital, 75100 Matera, Italy; giuseppe.trojano@policlinico.ba.it

**Keywords:** Follitropin delta (Follitropin-Δ), Follitropin delta overdose, side effects of FSH

## Abstract

Follitropin delta (Δ) is a recombinant human follicle-stimulating hormone (rFSH), like natural human FSH, that can stimulate the development and growth of multiple follicles. Treatment with Follitropin-Δ may cause mild to severe adverse reactions, such as the risk of developing ovarian hyperstimulation syndrome, resulting in nausea, vomiting and diarrhea, weight loss, respiratory difficulty, stomach swelling and discomfort of the pelvic area, headaches, and fatigue. To date, the effects of a Follitropin-Δ overdosage are unknown, and no data are reported in the scientific literature or in the drug data sheet. Therefore, this study aimed to describe the effects of Follitropin-Δ overdosages in poorly responding women who underwent IVF cycles. This is a descriptive case series of four nulligravid, poorly responding patients, two of whom made requests for fertility preservation. Four poorly responding patients who were prescribed 20.0 µg/day of Follitropin-Δ for three consecutive days wrongly injected the total cartridge of 72 µg Follitropin-Δ every day. After the incorrect injection of Follitropin-Δ, the patients continued their controlled ovarian stimulation and underwent vaginal ovarian pick up. The analyzed patients had no side effects or adverse reactions. The evaluations reported in this case series showed that the accidental use of 72 µg/day of Follitropin-Δ for three days did not cause side effects or adverse reactions in poor responders.

## 1. Introduction

Women undergoing controlled ovarian stimulation using assisted reproductive technologies (ARTs) require the administration of gonadotropins, such as a recombinant human follicle-stimulating hormone. Follitropin delta (Δ) (Rekovelle Subcutaneous Injection 12 μg/36 μg/72 μg Pen) has recently entered the pharmacological market for controlled ovarian stimulation in women undergoing ARTs. It is a recombinant human follicle-stimulating hormone (rFSH) developed by Ferring Pharmaceuticals Co., Ltd. (Saint-Prex, Switzerland). It is produced into a human-derived cell line of PER.C6 with an α2.3 and α2.6-linked sialic acid sugar chain [1]. The amino acid sequences of the two FSH subunits α and β are identical to the endogenous human FSH sequences with α2,6-linked sialic acid and bisecting N-acetylglucosamine, which are not present in Follitropin-α and Follitropin-β. So Follitropin-Δ has different glycosylation, pharmacokinetics, and pharmacodynamics from other follitropins. It shows a long half-life and low clearance from the serum that is related to the hepatic sialo-glycoprotein receptor (ASGPR) that contributes to the different pharmacokinetics and efficacies of therapeutic protocols for infertility treatments with Follitropin-Δ [1]. The mechanism of action of Follitropin-Δ is related to the FSH receptor of the ovary, which triggers several intracellular responses for the development and growth of multiple follicles [2]. Follitropin-Δ is given by injection under the skin through a multidose pen injector [3]. Unlike other commercial follitropins, graduations on the Rekovelle injector are expressed in µg. Treatments with Follitropin-Δ should be initiated on day 2 or 3 after the start of menstrual bleeding and continue until adequate follicular development has been achieved. The daily dose of Rekovelle is individualized for each patient to reduce each woman’s risk of ovarian hyperstimulation syndrome, while not compromising her potential for a successful outcome. The individual daily dose must be maintained throughout the stimulation period, but it can be modified according to the patient’s ovarian response [4]. The individual daily posology is determined by a validated algorithm that is based on the woman’s body weight and the serum anti-Müllerian hormone (AMH, within the last 12 months) concentration measured by the following diagnostic tests: the ELECSYS AMH Plus immunoassay from Roche (Basilea, Switzerland), or alternatively, the ACCESS AMH Advanced from Beckman Coulter (Brea, CA, USA) or the LUMIPULSE G AMH from Fujirebio (Shinjuku-ku, Tokyo, Japan) [5,6,7,8,9,10,11,12,13].

There is relevant use of Follitropin-Δ for women who undergo ART procedures [14,15], regardless of their geographical area [16,17,18]. To date, it has been used for normo-responder or hyper-responder patients, but recent studies also indicate increasing use of Follitropin-Δ for controlled ovarian stimulation of poorly responding patients. According to the Bologna criteria, the term poorly responding patients refers to a group of patients with a poor prognosis due to their low ovarian reserve. In fact, low ovarian reserve is associated with extremely low oocyte retrieval and embryonic production, so much so that in some cases, it is difficult to even transfer an embryo. It has been demonstrated that administering Follitropin-Δ daily results in a higher ovarian response (e.g., estradiol and inhibin B serum concentrations and follicular volume) compared to Follitropin-α administration in equal IU doses [19]. Follitropin-Δ administration seems to be the most suitable to improve the recruitment of the follicular cohort in poorly responding patients.

Treatment with Follitropin-Δ can be associated with side effects that must be considered. In detail, the treatment with Follitropin-Δ may cause mild to severe adverse reactions, such as nausea, vomiting and diarrhea, weight loss, respiratory difficulty, stomach swelling and discomfort of the pelvic area, headaches, and fatigue. The clinical picture may be complicated by thromboembolic events or excessive ovarian enlargement with consequent ovarian torsion [18,20,21]. In Table 1, the adverse effects reported to date are listed and classified by organ class and frequency.

However, it must be underlined that these mild or moderate risks have been commonly reported and should be considered as an intrinsic risk of the stimulation procedure and are linked to the ovarian reserve. Moreover, it seems that Follitropin-Δ, compared to the other follitropins, is able to partially prevent these symptoms [21].

To date, the effects of Follitropin-Δ overdosage are unknown, and no data are reported in the scientific literature or in the drug data sheet.

Therefore, the aim of this study was to describe the effects of Follitropin-Δ overdosages during the controlled ovarian stimulation of poorly responding women who underwent ART procedures to perform fertility treatments or oocyte cryopreservation.

## 2. Case Series Presentation

This is a descriptive case series of four nulligravid, poorly responding patients who were treated with Follitropin-Δ (Rekovelle, Ferring Pharmaceuticals Co., Ltd., Saint-Prex, Switzerland) for controlled ovarian stimulation at a private infertility clinic in Italy to perform IVF treatment or fertility preservation by oocyte cryopreservation. About 630 IVF cycles per year are carried out at the clinic. Before enrolling in IVF procedures, all patients underwent detailed standard investigations, and their tests were considered normal (Table 2).

There were about 90 patients who were prescribed Follitropin-Δ for ovarian stimulation in our clinic during the last two years since the equivalence of Follitropin-Δ to the other gonadotropins was demonstrated in terms of response and treatment outcomes [22,23,24,25]. The maximum daily dosage for the first treatment cycle normally does not exceed 12 micrograms. However, there is an increasing scientific background that makes the Follitropin-Δ starting dose calculated by the algorithm inappropriate. Arce et al. [26] demonstrated that daily Follitropin-Δ doses of 10 µg provide a similar ovarian response to 150 IU/day of Follitropin-α in IVF/ICSI patients, and so 20 µg of Follitropin-Δ would be expected to provide a comparable ovarian response to the maximum administrated dose of 300 IU/day of Follitropin-α. Moreover, it has been demonstrated that the administration of daily doses of Follitropin-Δ ranging from 12 to 24 µg for healthy Chinese women were well-tolerated [27,28].

Given these considerations, in our clinic, women with poor ovarian response and very low AMH (<1.00 ng/mL) and antral follicle count (AFC) values were treated, only after prior consultation and obtaining a signature of informed consent, with 20 µg/day of Follitropin-Δ.

During the last two years, four poorly responding patients who were prescribed 20 µg/day of Follitropin-Δ for three consecutive days wrongly injected the total cartridge of 72 µg of Follitropin-Δ every day. After the incorrect injections of Follitropin-Δ, patients continued the controlled ovarian stimulation and underwent transvaginal ovum pick up (OPU) for IVF procedures or oocyte cryopreservation.

### 2.1. Case 1

A 36-year-old nulligravid woman was seen for the first consultation on 9 May 2019 for long-term infertility (more than 1 year of trying to conceive). This patient weighed 79 kg with a body mass index (BMI) of 24.4. The patient applied for fertility preservation, although it was clearly shown that the clinical conditions did not make the attempt worthwhile. The patient still wanted to carry out the treatment. The male partner underwent semen analysis (spermiogram evaluation), color Doppler ultrasonography of the scrotum, and hormonal level dosages, which all turned out to be normal.

#### Medical History of the Patient

The patient was born in a eutocic delivery and had childhood exanthematous diseases. Her menarche was at 12 years of age, and her menstrual cycles were regular, lasting 3 days with an interval of 26 days. Her family’s and her medical histories were unremarkable, and she was not on any medications. The patient had allergic diathesis. At the age of 22, she underwent osteosynthesis surgery for a fracture of the right tibia after a road accident. After the incorrect administration of Follitropin-Δ, the patient continued the stimulation cycle, and four metaphase II oocytes were retrieved and then cryopreserved.

### 2.2. Case 2

A 41-year-old nulligravid woman was seen for her first consultation on 3 July 2019. At that time, the patient had been diagnosed with a low ovarian reserve and had been trying to conceive for one year. This patient weighed 79 kg with a BMI of 26.9. The male partner underwent semen analysis (spermiogram evaluation), color Doppler ultrasonography of the scrotum, and hormonal level dosages, which all turned out to be normal.

#### Medical History of the Patient

The patient was delivered by cesarean section and had childhood exanthematous diseases. Her menarche was at 12 years of age, and her menstrual cycles were regular, lasting 3 days with intervals of 26 days. Her medical history was unremarkable, but she had a family medical history of early menarche. She did not have allergic diathesis; she was not on any medications and had never had any surgery. After the incorrect administration of Follitropin-Δ, the patient continued the stimulation cycle, and four oocytes were recovered; three of them were found at metaphase II and injected with ICSI. One embryo was obtained and transferred on day three. The pregnancy test came back negative (Table 3).

### 2.3. Case 3

A 35-year-old nulligravid woman was seen for her first consultation on 25 May 2021 after four years of infertility history. This patient weighed 64 kg with a BMI of 22.9. The patient applied for fertility preservation, although it was clearly shown that the clinical conditions did not make the attempt worthwhile. The patient still wanted to carry out the treatment. The male partner underwent semen analysis (spermiogram evaluation), color Doppler ultrasonography of the scrotum, and hormonal level dosages, which all turned out to be normal.

#### Medical History of the Patient

The patient was born in a eutocic delivery and had childhood exanthematous diseases. Her menarche was at 12 years of age, and her menstrual cycles were regular, lasting 5 days with an interval of 28 days. Her family medical history was unremarkable, and she did not have allergic diathesis. At the age of 32, she was treated for a psychiatric disorder. The patient had a history of endometriosis (stage IV), for which she had laparoscopic surgery in June 2019. Two endometriotic ovarian cysts were removed during the surgery. At the time, she was not on any medications. After the incorrect administration of Follitropin-Δ, the patient continued the stimulation cycle, and three metaphase II oocytes were retrieved and then cryopreserved (Table 3).

### 2.4. Case 4

A 38-year-old nulligravid woman was seen for her first consultation on 1 June 2021. This patient weighed 55 kg with a BMI of 19.5. At that time, the patient had been diagnosed with a low ovarian reserve and had been trying to conceive for four years, with a history of unexplained recurrent spontaneous miscarriage. The male partner underwent semen analysis (spermiogram evaluation), color Doppler ultrasonography of the scrotum, and hormonal level dosages, which all turned out to be normal.

#### Medical History of the Patient

The patient was born in a eutocic delivery and had childhood exanthematous diseases. Her menarche was at 11 years of age, and her menstrual cycles were regular, lasting 3 days with intervals of 25 days. Her family’s and her medical histories were unremarkable, and she was not on any medications. She was without allergic diathesis, and she underwent two laparoscopic surgeries, during which multiple myomectomies were performed. After the incorrect administration of Follitropin-Δ, the patient continued the stimulation cycle. Just one metaphase II oocyte was recovered and, after fertilization by ICSI, the obtained day-three embryo was transferred. The pregnancy test came back negative (Table 3).

The clinical team, after learning about the incorrect administration of Follitropin-Δ, invited the patients to the center. Patients were subjected to anamnesis, clinical examinations, and blood tests to check their state of health and evaluate any medical side effects or adverse events. All patients had normal vital signs, heart rhythms, and blood pressure. The blood tests and electrocardiograms were all normal. Coagulation factors and liver and kidney functions were also normal. Only two patients presented with mild nausea and headaches. All the data collected are reported in Table 4.

After verifying that none of the patients had suffered any adverse reactions or severe side effects, stimulation was continued with daily versus alternate-day monitoring. Every time the monitoring was performed, complete blood chemistry tests and instrumental evaluations were checked, and they always turned out to be normal.

## 3. Methods

This retrospective case series study was based on data from four case reports of poorly responding women who were treated with Follitropin-Δ (Rekovelle Subcutaneous Injection 12 μg/36 μg/72 μg Pen) to induce ovulation according to standard practice criteria. For three consecutive days, these patients injected 72 µg of Follitropin-Δ instead of 20 µg/day.

After identifying the incorrect drug administration, patients underwent anamnesis; blood test evaluations to check their estradiol and progesterone levels, coagulation factors, and liver and kidney function; electrocardiography; blood pressure examinations; and abdominal ultrasonography.

Patients continued the controlled ovarian stimulation with 20.0 µg/day of Follitropin-Δ. In the last four days of the stimulation cycle, 0.25 mg/day of Ganirelix (Fyremadel, Ferring Pharmaceutical, Saint-Prex, Switzerland) was added. Between 34 and 37 h before oocyte collection, patients received 5000 IU of Gonasi (IBSA, Lugano, Switzerland).

Transvaginal ovum pick up (OPU) was performed under ultrasound guidance (VOLUSON S8, GE Healthcare, Chicago, IL, USA) [29]. The retrieved cumulus–oocyte complexes were denuded, and metaphase II (MII)-stage oocytes were selected under a stereomicroscope (Nikon SMZ 1500, Tokyo, Japan) and injected using the ICSI technique [30,31,32] (for patients undergoing IVF treatment) or cryopreserved (for patients undergoing fertility preservation procedures despite the low values of AMH and AFC). After ICSI, embryos were cultured in the Geri time-lapse incubator (Genea Biomedx, Sidney, Australia) by using a single-step medium (GTLTM, Vitrolife, Gothenburg, Sweden). Embryonic development was monitored daily, and on day 3, embryos were transferred [33]. Pregnancy was confirmed by serum levels of beta-human Chorionic Gonadotrophin (beta-hCG) exceeding 15 mIU/mL on the 12th day after transfer. Oocyte cryopreservation was performed by using the vitrification Cryotop method for the oocytes as per the manufacturer’s instructions (Kitazato, Tokyo, Japan).

## 4. Discussion

In recent years, there has been increasingly widespread use of Follitropin-Δ in many IVF Centers. Although it was first used to treat normo-responding or hyper-responding patients, in recent years, increasing use of Follitropin-Δ to perform ovarian stimulation in poor responders has been recorded. The maximum recommended dose is 12 µg/day. However, different studies have shown the limitations of this dosage for patients with poor ovarian response [26]. Given the evidence that 150 IU of Follitropin-α corresponds to 12 µg of Follitropin-Δ, the daily dosage of Follitropin-Δ for poor responders could be identified as 24 µg/day. Despite the increasingly widespread use of this molecule, so far, no research studies have been performed to investigate the side effects of Follitropin-Δ overdosages.

This case series described the prolonged effects of the administration of 72 µg/day of Follitropin-Δ for three consecutive days in patients with poor ovarian response. Four patients who were prescribed 20 µg/day of Follitropin-Δ for three consecutive days, instead of administering the prescribed dose, injected the entire 72 µg pen every day. At the check, once the incident was discovered, they were kept under observation. The analyzed patients had no severe side effects or adverse reactions, neither the day after the three incorrect administrations nor during the whole stimulation. So, they underwent normal OPU and fertilization by the ICSI technique or cryopreservation. For patients underwent in vitro fertilization treatment cycles, fresh embryo transfer was performed on day three without reporting any pregnancy. However, pregnancy failure may have arisen due to the very low ovary reserve of both patients.

An even more important discovery emerged from the careful analysis of the patients after they made the mistake: none of the four patients reported significant side effects or adverse reactions, with the exception of two patients who experienced mild nausea and headaches. Moreover, apparently the excessive introduction of the drug did not in any way alter the subsequent course of the ovarian stimulations, which came to the oocyte retrievals normally. Furthermore, both of the patients who asked for fertility preservation froze the oocytes, and the others managed to get to the transfer, although without getting pregnant. It should, however, be noted that, objectively, both the cryopreservation of a few oocytes and the absence of pregnancy can be correlated with the extremely deficient clinical picture presented by all the patients described rather than the errors the patients made during the ovarian stimulation.

The most important Follitropin-Δ side effects, like for the other follitropins, can be generated if there is a follicular response that generates a good elevation of estradiol levels. Our data clearly show that the estradiol levels of the patients under stimulation did not reach values above 750 pg/mL, while the progesterone values remained within the norm, i.e., below 1.2 ng/dL.

Like all molecules, in addition to having pharmacokinetic effects related to its activity, there are effects that are related to non-main activities of the molecule. Also, in this case, the patients reported no side effects or important adverse reactions.

This study is limited by a sample consisting of only poorly responding patients that, notoriously, are less responsive to FSH therapy and normally require higher doses of follitropin than are typically needed to respond to stimulation. No data are available about the effects of Follitropin-Δ overdosages on normo- or hyper-responders to confirm its harmlessness, considering also that a higher daily amount of Follitropin-Δ was suggested by some authors [28,34].

## 5. Conclusions

The evaluations reported in this case series showed that the accidental use of 72 µg/day of Follitropin-Δ, which is higher than the normally prescribed doses, for three days apparently did not cause severe side effects, except for two patients who had mild headaches and nausea, and there were no adverse reactions, at least in the poor responders. Moreover, despite the incorrect Follitropin-Δ administration, all the patients underwent OPU, and matured oocytes were recovered and were frozen or were able to be normally fertilized in vitro and produce live embryos to be transferred. These are the first cases of Follitropin-Δ overdose described in poor responders. There are still no data available in the case of overdoses in normal-responding or hyper-responding patients, in whom the follicular response could be quite different.

## Figures and Tables

**Table 1:** Adverse reactions to Follitropin-Δ in patients who underwent ART procedures [18,20,21].

Organ Class	Common	Uncommon
Psychiatric disorders and nervous system disorders	Headache	Mood swings, dizziness, and drowsiness
Gastrointestinal pathologies	Nausea	Diarrhea, vomiting, abdominal discomfort, and constipation
Reproductive system diseases and mammary pathologies	Discomfort of the pelvic area and uterine appendages	Vaginal bleeding, breast tenderness and pain
General disorders and administration site conditions	Fatigue	

Abbreviation: ART = assisted reproductive technologies.

**Table 2:** Summary of patients’ pretreatment characteristics.

	Case 1	Case 2	Case 3	Case 4
Age (yrs)	36	41	35	38
Body mass index (BMI)	24.4	26.9	22.9	19.5
Diagnosis	Reduced ovarian reserve Fertility preservation	Reduced ovarian reserve	Reduced ovarian reserve Fertility preservation	Recurrent miscarriages
Parity	0/0/0/0	0/0/0/0	0/0/0/0	0/0/3/0
Blood type	0+	0+	0+	0+
Antithrombin III %	91	91	91	91.3
C-Protein %	85	85	85	97
S-Protein %	94	94	94	55
Anti-Cardiolipin IgG	Normal	Normal	Normal	Normal
Lupus anticoagulants	Normal	Normal	Normal	Normal
Factor V Leiden	Normal	Normal	Normal	Normal
Factor II	Normal	Normal	Normal	Normal
TSH	0.87	2.01	1.62	1.84
Basal E2 (pmol/L)	81	101	72	58
Basal FSH (IU)	11.6	14.6	9.2	8.7
Basal P4 (nmol/L)	0.35	0.24	0.42	0.37
Antral follicle count (AFC)	8	6	7	3
AMH	0.64	0.19	0.75	0.11
Vitamin D	28	28	28	32

Abbreviations: TSH = thyroid stimulating hormone; E2 = estradiol; FSH = follicle stimulating hormone; AMH = Anti-Mullerian Hormone.

**Table 3:** Reproductive treatment outcomes.

	Case 1	Case 2	Case 3	Case 4
Date of stimulation	28 June 2019	5 September 2021	21 June 2021	30 June 2021
Data fine stimulation	6 July 2019	16 September 2021	3 July 2021	14 July 2021
Total FSH (µg)	336	406	406	436
Number of follicles at the end of stimulation (n)	4	5	4	3
Endometrium thickness (mm)	8.4	8.5	8.1	7.9
Number of oocytes retrieved (n)	4	4	4	1
Number of MII oocytes (n)	4	3	3	1
Number of MII oocytes cryopreserved	4	-	3	-
Number of embryos obtained (n)	-	1	-	1
Number of embryos transferred (n)	-	1	-	1
Pregnancy	-	No	-	No

Abbreviation: FSH = follicle stimulating hormone.

**Table 4:** Complete hematic–chemical and instrumental examinations after Follitropin-Δ overdosages.

	Case 1	Case 2	Case 3	Case 4
Serum Estradiol (pg/mL)	261	165	89	104
Serum Progesterone (ng/mL)	0.75	0.81	0.62	0.71
Abdominal ultrasonography	Normal	Normal	Normal	Normal
Blood pressure (mmHg)	130/85	110/75	125/85	120/80
Electrocardiography	Normal	Normal	Normal	Normal
GOT and GPT (U/L)	23–28	25–29	22–28	25–27
Alkaline phosphatase (U/L)	160	180	172	155
Azotemia (mg/dL)	40	39	44	42
Creatininemia (mg/dL)	0.93	0.91	0.87	0.83
Glomerular filtration rate (mL/min)	95	92	94	90
Blood sugar (mg/dL)	89	82	92	91
PT (Inr)	1	0.97	0.98	0.95
PTT (s)	32	32	34	31
Antithrombin III (%)	94	89	97	92
D-dimer (ng/mL)	410	387	390	381
Side effects	Mild headache	Mild nausea	-	-
Adverse reactions	-	-	-	-

Abbreviation: GOT = Glutamic-Oxalacetic Transaminase; GPT = glutamic pyruvic transaminase; PT = prothrombin time; PTT = partial thromboplastin time.

## Data Availability

All data are reported in the text.
